# Linkage of health and aged care service events: comparing linkage and event selection methods

**DOI:** 10.1186/1472-6963-8-149

**Published:** 2008-07-17

**Authors:** Rosemary Karmel, Diana Rosman

**Affiliations:** 1 Australian Institute of Health and Welfare, Canberra, Australia; 2 Data Linkage Branch, Department of Health Western Australia, Perth, Australia

## Abstract

**Background:**

Data linkage is a technique that has long been used to connect information across several disparate data sources – most commonly for medical and population health research. Often the purpose is to connect data for individuals over extended time periods or across different service settings, and so person-based linkage using detailed personal information is preferred. Linkage which aims to link connected events, on the other hand, requires information about the time and place of the event as well as the person or persons involved in that event in order to make high quality linkages.

This paper describes the detailed process of event linkage and compares directly an event-based linkage method for identifying transition events between two care sectors in Australia with a well-established high quality longitudinal person-based linkage which facilitates identification of event data for individuals.

**Methods:**

Direct comparisons are made between transition events identified using an event-based linkage and an existing person-based linkage for people moving from hospital into aged care in Western Australia. Several aspects of event-based linkage are examined: refinement of the strategy to reduce false positives, causes of false positives and false negatives, quality of the linked event dataset, and utility of the linked event dataset for transition analysis.

**Results:**

Over 97% of the event-based links were among those selected using the person-based linkage and over 90% of the latter were identified by the event-based method, with the remainder missed mostly due to differences in reported event date or residential region. Consequently the two linked datasets were sufficiently similar to give very similar results for analyses, but the actual volume of movement from hospital to RAC was underestimated by about 10% by the event-based method.

**Conclusion:**

This project has allowed a 'preferred event' event-based linkage strategy to be selected and deployed across Australia to study movements from hospital to residential aged care facilities using databases in which only limited personal information is available, but event dates and details can be readily accessed. The utility of this approach in other transition situations depends on the volume of movement and the accuracy of recording information in each setting.

## Background

Data linkage is a technique that has been used since the 1940s to connect information across several disparate data sources, most commonly for medical and population health research. The technique uses as much information as is available in each data source to join records or other data structures that are thought to belong to the same person, place or event. Person-based linkages use personal information such as name, address and date of birth, or a personal identification number, to connect data for individuals over extended time periods or across different service settings. However, linking events for people requires information about the time and place of the event as well as the person or persons involved in that event to make high quality linkages. Additional details that might be required for an event linkage are the location of the event (i.e. the establishment or address) as well as the time and date when the event occurred.

The quality of any linkage relies on the amount of information available for linkage as well as that information's accuracy, consistency and completeness. For instance, person-based linkages across a long time period require access to a universal, unique identifying numbering system or extensive personal details such as name, address and date of birth. However, within a narrow period of time or limited population group, fewer details may be needed to achieve links of the same quality.

Event linkages may be achieved through reference to the event details in isolation, or may be achieved by selecting specific events from a sequence of events within, say, a person's linked health history. Some events, such as environmental accidents and road crashes, can involve more than one person and rely heavily on the time and place of the event to make the connection between affected individuals. On the other hand, a person may have several similar related 'transition' events occurring within a limited period of time, for example movement between one health setting and another. Identification of these related events requires information about the individual as well as the time and place of the event. An event sequence may occur over a period of time that may be seconds, minutes, hours or days.

An important example of a transition event is when a person moves from hospital into an aged care facility. In Australia, where 13% of the population of 20.5 million is aged 65 or over and 5% of these are in residential aged care [1,2], there is considerable movement between the hospital and residential care sectors by older people. With an estimated 7–8% of older people in hospital on any one night having their usual residence in an aged care home and perhaps as many as 50% of admissions into such homes coming from hospital [3,4] movement between the two sectors is becoming an important issue for Australia, as it is in other ageing societies. Consequently, understanding the transition between hospital care and residential aged care (RAC) is important in aged care service delivery (see, for example, [5-9]).

Australia-wide administrative data are available separately for hospital and RAC episodes. These data provide little information on the movements of clients between the residential and acute care sectors, and the two datasets contain neither a common person identifier nor detailed identifying information (such as name). However, a range of data items is common to both datasets, and event linkage, without access to comprehensive personal information, could be used to provide insight into transitions between hospital and RAC. Such linkage has been used successfully in a number of other scenarios [10-12]. These studies commonly include linkage carried out using event dates, establishment location(s) in conjunction with other event-specific variables as well as the individual's date of birth and sex.

Two earlier studies suggested that there is adequate personal demographic information in conjunction with event information available for linking the transition events on the two Australian datasets. Theoretical investigations have shown that the expected rate of false matches is low [8] and, taken together with an empirical feasibility study [13], suggest that the set of linked client records resulting from the event-based identifier-free linkage strategy could provide a useful resource for examining service use patterns associated with movements between the two sectors.

While the earlier studies point convincingly towards the utility of the event-based linkage strategy, the absence of validation of the resulting links against a reference standard still leaves room for doubts. Investigations of linkage validity are rare [14], and comparisons of linkage strategies uncommon [15]; however, available assessments of matching strategies have shown that linkage with limited personal information can be useful in a number of areas [10-12,16-18]. This paper examines this issue for health service transition events through direct comparisons of independently conducted linkage strategies using identical datasets. In particular, using the specific example of transition from hospital into RAC, this paper describes the detailed process of event linkage and compares a 'preferred transition' event-based linkage method directly with transition events identified using a well-established high quality longitudinal person linkage with access to a 40 year archive of comprehensive personal identifying information and associated event data. Although any selection of events from among the chains of linked events for individuals is not guaranteed to be error-free, this set of person-based links is taken as the 'gold standard' for this project.

## Methods

In the current analysis, we compare two distinct record linkage strategies for unidirectional linking of discharges from hospital with the corresponding admissions into or returns to care in a RAC facility. The scope of the comparisons is limited to movements from hospital to RAC by people aged 65 and over in Western Australia during 2000–01. Western Australia was chosen for this study because of its unique position in Australia of having a well-established health data linkage system that includes both hospital and RAC data [19]. Overall, in Western Australia in 2000–01 there were just over 86200 hospital discharges for people aged 65 and over (excluding same-day episodes and those ending in a within-sector transfer). For RAC, there were 19600 entries and re-entries into RAC for older people, with about 40% of these relating to admissions into RAC for either permanent or respite care, 35% being for permanent aged care residents temporarily in hospital (termed hospital leave, and including some for people who were discharged to hospital or who died while in hospital), and the remaining 25% being for permanent residents on social leave with family and/or friends – a small number of whom may have had an episode of hospitalisation during their leave period.

### The linkage strategies

Two independent linkage processes were used in this study. The first linkage strategy was a selection process from an existing person linkage – here termed N linkage – carried out by the Data Linkage Branch of the Western Australian Department of Health. The second was the event-based E linkage strategy developed within the Australian Institute of Health and Welfare (AIHW) [8,13] which matches event records using event dates and characteristics as well as date of birth, sex and geographic region of usual residence of the person involved. These two processes are outlined below. Readers requiring more detailed technical information on the linkage methodologies are referred to Karmel and Rosman 2007 [20].

This project was performed as part of the Western Australian Cross Jurisdictional Data Linkage Project covered by a Memorandum of Understanding between the Western Australian state Department of Health and the Australian Government Department of Health and Ageing. The person-based linkage was covered by approvals from the two agencies' confidentiality and ethics committees (approvals CHIC #200318 and DEC 3/2003, respectively). Before the event-based data linkage was undertaken, ethics approval was also obtained from the AIHW Ethics Committee (approval EC 305) and permission to use the required data was obtained from the relevant bodies.

### Selection from person (N) linkage

The person-based linkage was undertaken using the Western Australian Data Linkage System (WADLS), a dynamic ongoing system used to create and store a web of links across population and research health data collections in Western Australia. The system is updated continuously by a small number of dedicated staff. The WADLS probabilistic linkage process, which follows the 'Two-Stage Protocol' to protect privacy [21], uses as much personal information as is available to create and load links as they are discovered. Links between a large variety of health-related datasets are established and maintained in this system [19], including datasets on births, deaths and marriage registrations, electoral records, hospital episodes, midwives notifications, emergency presentations, mental health contacts, and cancer registrations, with some datasets dating from the 1970s. Most recently linkages to Medicare enrolments and aged care datasets including RAC have been included. The person-based linkage process, which includes an extensive clerical review phase, benefits from the availability of this archive of linked changes to personal and demographic details over up to 40 years. For many of the datasets covered by WADLS, including the hospital data, events information is available for use in linkage identification. However, RAC event information was not available for the WADLS person linkage process due to conditions set by the data provider.

The linked data represented within the WADLS varies from one single record to more than 3000 for persons either resident in Western Australia or receiving a service in Western Australia. As stated above, the linked records in the WADLS provide name and address reporting histories across a range of health service events over four decades resulting in linkage accuracy and completeness levels that are further improved with each new dataset that is incorporated. The N-linkage between hospital and RAC records also benefited from detailed clerical review using these resources.

The underlying quality of the person-based linkage in the WADLS has previously been measured by manually checking a sample of linked hospital admission and death records. The measured error rate of this section of the WADLS in 2001 and 2002 was less than 0.1% of persons having at least one incorrect event linked within their medical history. An earlier estimate (1998) of false negative errors estimated these at approximately the same level (0.1%) [19].

In order to select the events of interest for this study, all relevant hospital and RAC event records were retrieved separately from their original sources and then joined using relevant WADLS hospital-RAC person 'linkage keys'. From among this subset of linked records, the most appropriate hospital-RAC event link was chosen by measuring the closeness in time of the hospital and RAC event dates. For those individuals where there was a choice between different RAC event types, the match to the earlier RAC event was selected.

Overall, this N strategy resulted in 8106 links between hospital separations and entry (or re-entry) into RAC during the period of interest. Because of the high level of the accuracy of person links, these links were used as the reference linkage in this study.

### Event-based (E) linkage

Event-based E linkage strategies link records by using event dates and event characteristics information in conjunction with limited demographic data. The purpose of this strategy is to allow identification of related events for people when there is insufficient non-event information to establish person links as a precursor to identifying related events. The resulting linked dataset may vary depending both on how much of this information is used when identifying links, and how particular data items are used to specify the linkage process.

To make the most of the availability of a reference dataset (i.e. N links) a step-wise approach was taken to comparing match results and refining the E linkage strategy:

• Match using a specific version of E linkage

• Compare results to N linkage

• Identify shortcomings

• Consider options for improvement

• Test proposed improvement.

### Initial strategy

Basic E linkage – as used in the feasibility study [13] – was initially used to establish the accuracy of the simplest form of E linkage in the current context. Under this strategy, all hospital and RAC events, irrespective of their characteristics, were matched on the single hospital exit/RAC entry transition date and the date of birth, sex, and region of usual residence of the client. Statistical local groups (SLGs, derived from a concordance between local government administrative regions and postcode) were used as the match region.

### Constrained strategy

The initial E linkage (as above) was applied using a single transition date and without taking into account how the two data collections were derived or whether there was other information that could be used to improve the matching process. The two datasets did in fact contain a range of information about their respective events. Variables on the datasets that could assist in isolating preferred transitions were identified by considering the actual relationship between the hospital and RAC events and ascertaining which, if any, variables on the two datasets might help to describe this relationship. Questions considered to identify variables that could assist in the linkage process were as follows:

(a) A hospital episode has a start and an end date, with the latter being the transition date for hospital-RAC transition events. Are there additional event date data on the RAC dataset that relate to the hospital episode dates?

(b) Is there any information on the hospital data which indicates how the hospital event started or which sector the patient came from?

(c) Is there any information on the hospital data concerning where the patient went after discharge?

(d) Is there any information on the RAC data concerning where a resident was just prior to admission?

(e) Is it possible for there to be more than one event validly reported for the same person on the same day – either for hospital discharge events or RAC entry events?

(f) It is not clear from data definitions whether address of usual residence as reported in the hospital data is meant to refer to before the period in hospital or after. Is there any additional address-type information on either dataset that could assist in identifying events for people who change their usual residence on leaving hospital?

Answers to these questions led to identifying several additional data items on both the hospital and RAC datasets that could be used to support the E linkage (Table 1). The adjustments made to the basic E linkage strategy to incorporate this information in effect 'constrained' the linkage process through knowledge of both the service systems and data collection practices with a view to finding the best match using all available event date information and event descriptors.

**Table 1:** Identifying additional data for E linkage.

**Question 1**	**Are there additional event date data on the RAC dataset that relate to the hospital episode dates start and end?**
Findings	Events for people on leave from permanent RAC (either for hospital leave or for social leave) have both start and end dates recorded.
E strategy adjustment	Matches to the admission, hospital leave and social leave events were carried out separately, and start and end dates for hospital and social leave events were used when matching to hospital discharges.

**Question 2**	**Is there any information on the hospital data which indicates how the hospital event started or from which sector the patient came?**

Findings	Information on usual residence (private dwelling, RAC etc.) is not reliably available on the hospital dataset. A variable on hospital 'mode of admission' shows whether or not the hospital event started with a within-sector transfer or as an admission into hospital.
E strategy adjustment	Hospital mode of admission data influenced the type of date comparisons made when matching to RAC leave events.

**Question 3**	**Is there any information on the hospital data concerning where the patient went after discharge?**

Findings	'Mode of discharge' is reported on the hospital data. Categories include whether the patient transferred within the sector, died in hospital, returned to their usual residence or went to RAC. Death in hospital is well-reported but return to RAC as the usual residence and admission to RAC are not well-distinguished.
E strategy adjustment	Hospital episodes ending with a within-sector transfer were excluded from the linkage process (also done for the basic E linkage). Hospital discharges were divided into three groups defined by reported destination on discharge: 'to usual residence', 'died' and 'other' (predominately people reported as discharged to RAC). The three groups of RAC events (admissions, hospital leave and social leave) were then matched separately to each of these three groups, with the exception that RAC admissions were not matched to hospital discharges due to death. This reduced the incidence of within-dataset duplicates with respect to match variables and so allowed greater flexibility in choice of match variables.

**Question 4**	**Is there any information on the RAC data concerning where a resident was just prior to admission?**

Findings	The RAC dataset does not explicitly contain information about a client's location just prior to admission. However, data on place of assessment for aged care (including whether in hospital) and date of the assessment are available.
E strategy adjustment	Place and date of aged care assessment were used to assist linkage when matching using hospital region (see 6. below)

**Question 5**	**Is it possible for more than one event to be reported for the same person on the same day – either for hospital discharge events or RAC entry events?**

Findings	Hospital episodes may last less than a day (same-day episodes), so a person may have two hospital episodes starting or ending on the same day. In addition, people may go to a different RAC facility at the end of their hospital leave. Such a change in RAC facility is recorded both as a return from hospital leave and as an admission into RAC, resulting in two entry events reported for the same day relating to the same event (i.e. collision events).
E strategy adjustment	Because same-day hospital episodes are highly unlikely to be related to a RAC entry event, same-day hospital episodes were excluded from the linkage process (also done for the basic E linkage). To select between any duplicate matches arising from multiple events on the same day for the same person or multiple comparisons between the groups of RAC events and hospital discharge groups, likely matches from the various comparisons were given a priority ranking. Priority ranks were based on reported destination on discharge from hospital, in addition to a preference for matches to a RAC hospital leave record over a RAC admission record (as a link to the former indicates that the person was already living in RAC), with matches to social leave being least preferred.

**Question 6**	**Is there any additional address-type information on either dataset that could assist in identifying events for people who change their usual residence on leaving hospital?**

Findings	Other available address-type information includes postcode of the hospital and postcode of the receiving RAC facility.
E strategy adjustment	Usual residence was grouped into regions of various size to allow for movement within a specified neighbourhood. Hospital region and RAC facility region as well as reported region of the person's usual residence were considered for matching.

Adjustments to E linkage strategies based on knowledge of service systems and additional information in RAC and hospital data collections.

For both E strategies, matching was undertaken using probabilistic methods via *WebSphere*^® ^software. This statistical package contains a suite of tools for identifying duplicate records for individuals (or entities) within datasets and for matching records for the same individual (or entity) across datasets; *WebSphere*^® ^was previously known as *Automatch*^® ^and *Integrity*^® ^[22]. Probabilistic matching generally involves two types of data items: blocking and match variables, with match variables being compared only within categories of blocking variables (known as a pass) thereby greatly reducing the number of pair-wise comparisons needed to identify matches. Consequently, when identifying links, variables used in blocking must match exactly, while those used in matching can vary slightly. The degree of similarity in these match variables is assigned a match weight which is aggregated across all the variables being matched in a particular pass to arrive at a cumulative weight that is either accepted (high weight) or rejected (low weight) as a match – or link. Matches based on data of varying accuracy are identified by running multiple passes with different arrangements of blocking and match variables. For E linkage, matches identified in one pass were excluded before proceeding to the next pass.

For the current project, basic event characteristics were used to subset the datasets prior to matching to improve the likelihood of identifying appropriate links (see Table 1 for details). A subset of hospital discharge records was then matched to a subset of RAC entry records and a single (if any) match was selected based on the match weight. Table 2 summarises the general approach taken to blocking and match variable specification in the *WebSphere*^® ^matching process. In general, smaller regions were used in earlier passes to ensure that matches based on the most detailed information were identified first. Broader match regions were then used to allow for change of address within the neighbourhood (e.g. to live with relatives or to go into RAC near their previous usual residence). Match passes were undertaken in the order indicated in the table. If exact duplicate matches occurred in a pass, one was selected at random (this rarely happened). When event dates were used as match variables, match criteria were set within *WebSphere*^® ^specifying that a gap of no more than two days between hospital exit and RAC entry event dates was allowed when identifying matches. This approach was taken both to reduce the number of person-level false positives (i.e. matching events for different people) and to reduce within-person false positives (i.e. linking the wrong two events for the same person). A small gap was tolerated to allow for inconsistencies in reporting date on the two administrative systems. After matching using *WebSphere*^®^, a number of deterministic rules were applied to ensure that allowable differences in the values of the match variables were not violated.

**Table 2:** Blocking and matching variables used in probability matching passes for E linkage strategies

**Pass**	**Blocking variables**	**Match variables**
1	region definition 1 sex date of birth event dates	none
2	region definition 2 sex date of birth	event dates: one-sided variation (non-overlapping)
3	region definition 2 sex day of birth month of birth event dates	year of birth
4	region definition 2 sex year of birth event dates	day of birth month of birth
5	region definition 2 sex date of birth	event dates: two-sided variation
6	region definition 3 sex date of birth event dates	none
7	region definition 4 sex date of birth event dates	none

Note: Dates were only used as blocking variables if an exact date match was appropriate. Early investigations indicated that allowing variation in both date of birth and event dates leads to an unacceptable number of erroneous matches.

Detailed technical information on the E linkage process is contained in Karmel and Rosman 2007 [20]. To protect the privacy of individuals, the linkage was carried out by the AIHW using the Institute's protocol for linking between two or more data sets held within the AIHW [23].

### Comparing linkage strategies

When linking any pair of records four outcomes are possible: a true link (true positive), no link (true negative), a mis-link (false positive) and a missed link (false negative). In the event-based strategy, false positive links can be caused by several individuals – either leaving hospital or entering RAC – having the same basic demographic data so that events for the wrong people are linked together. In addition, errors or inconsistencies in the event information for the same person may cause both false positives and false negatives.

Given the high quality of the WADLS person-based links, N-linkage was used here as the reference standard against which the E linkage results were compared to determine whether an E link (or lack thereof) was 'true' or 'false'. Comparisons between N and E linkage results were undertaken both to measure the quality of E links and to identify causes of false positives and false negatives, thereby allowing refinement of the method for the current application.

In this paper, two key measures are used when comparing matches. Using terminology originating in epidemiology and clinical trials [24], these are the positive predictive value (PPV, the per cent of E links that are true links) and sensitivity (per cent of all links that are identified by the E linkage strategy). Specificity (E true non-links as a per cent of all non-links) and negative predictive value (NPV, the per cent of E non-links that are true non-links) are only briefly considered as their values vary depending on whether they are derived from the point of view of hospital discharges or RAC entries. The definition of these measures is illustrated in Table 3.

**Table 3:** Comparing E links with N links

	**N link**	**N non link**	
**E link**	a	b	PPV = a/(a+b)
**E non link**	c	d	NPV = d/(c+d)
	Sensitivity = a/(a+c))	Specificity = d/(b+d)	

Note: The number of non links depends on whether non links to hospital episodes or RAC events are being considered. Therefore the values of NPV and specificity are different depending on whether they are derived from the point of view of hospital discharges (86200 in total) or RAC entries (19600 in total).

**Figure 1:**
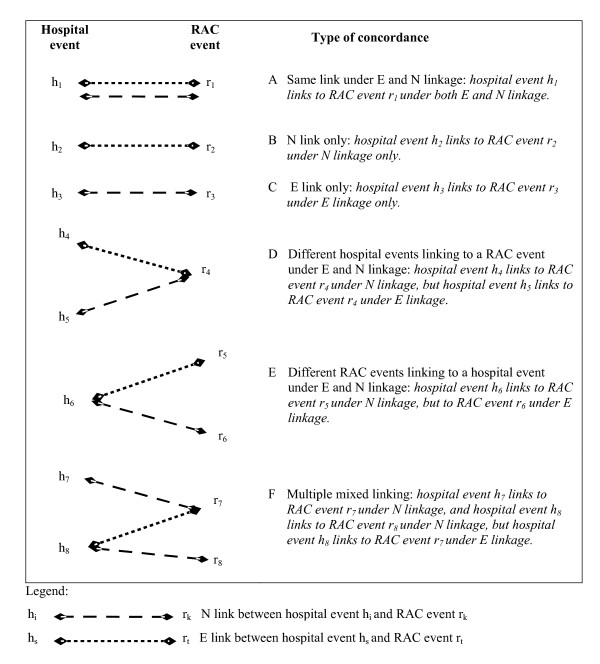
Types of link concordances between N and E links.

When comparing linkage strategies we can easily identify whether a record has been linked and whether it should have been linked. However, in the current context of identifying transition events it is also important that an event record in one dataset matches with the correct event record in the other dataset. That is, as well as knowing whether or not an event should have been linked, we need to know which event in the other dataset it should have linked to. Overall, as illustrated in Figure 1, there are six types of possible link concordances, including three (D, E and F) where there are both N and E links but different events are identified as the 'preferred transition' event by the two processes. Situations where matches of types D, E and F in Figure 1 should be considered true links or false links are discussed in the Results section.

### Refining E linkage

Direct comparison with a reference linkage provides a rare opportunity to refine a particular strategy by identifying those match passes that lead to too many false positives relative to true matches. By making such comparisons those passes that lead to an acceptably high likelihood of making a true match can be retained and those that have an unacceptably high likelihood of making a false match can be excluded. At the same time, passes whose exclusion would result in a high number of false negatives (that is, missed matches) can also be retained. It needs to be remembered, however, that the final strategy needs to be reproducible when there is no reference standard. One way of identifying an acceptable blocking/match pass strategy is to use the reference linkage to estimate the range of the actual PPV for a particular pass given the observed PPV. It is then assumed that in similar studies without a reference linkage similar PPVs would apply.

For the current study, from the point of view of ensuring a low rate of false positives, we wanted to be confident that, at the very least, a particular match pass resulted in more true matches than false matches. To this end, using standard statistical tests ([25] p82), the criterion used to identify poorly-performing matching algorithms was that the underlying PPV should be above 60% with 95% probability. Using this cut-off meant that, even if the actual PPV was as low as this, we would have made 50% more true matches than false matches under the strategy (60% versus 40%). Consequently, for E linkage strategies, when linking hospital and RAC datasets only those match passes in Table 2 with a PPV of at least 60% were retained for identifying event links.

## Results

The results of comparisons between matches identified through N linkage and those found using several E strategies are presented below. In addition, causes of false positives and false negatives are examined. Finally, to establish the overall utility of the E-linked data, we compare the results for three example analyses based on E and N linked datasets to see whether patterns within the various transition groups as identified through E linkage reflected those seen when using N linked data.

### Quality of the N linked dataset

The high quality of links in the N linked dataset was re-affirmed when reviewing the person links implied by the E-only links (i.e. those only found by the event-based method): E linkage found just two additional links between hospital and RAC clients that had previously been missed using the WADLS.

Selection of transition events from existing person links relies on two factors – the identification of the correct person (i.e. the assumption that the person links are correct) followed by selecting the correct pair or sequence of events for that person.

It may be possible to estimate false negative (or missed) links in some situations if there is an expectation of finding a matching transition record. Recorded hospital leave as a data item in the RAC dataset provided this opportunity: wherever the RAC facility recorded a visit to hospital, it would be expected that the person-based matching would result in hospital matches for (nearly) all such recorded RAC hospital leave events.

The N linkage strategy did not identify a hospital-RAC link for just over 4% of all RAC hospital leave events. This suggested that either

• the N strategy does not identify some people who really had used both RAC and hospital services, possibly due to insufficient or erroneous demographic details. The lack of event data on the RAC records for WADLS linkage may have contributed to this.

• the RAC or hospital administrative data systems contained reporting errors in event types or event dates preventing appropriate selection of event records among person records; or

• people may have left RAC to go to hospital but some were never actually admitted but were treated in an emergency department and so did not appear in the hospital 'admission' dataset.

A close examination of all the WADLS-linked events for emergency, hospital admission and death records for a sample of people with a link to RAC hospital leave missed by N linkage showed that all three of the above scenarios had occurred, with no one single cause dominating.

Taken together, the above results confirm the use of the N linkage as the reference linkage for this study.

### Accuracy of E linkage

For the initial basic E linkage strategy, only two match passes were used (passes 1 and 2 in Table 2) as comparisons with N linkage showed that passes allowing any further variation when matching led to PPVs of less than 55% and so were unacceptable. Basic E linkage identified 6693 event matches. Comparisons with N linkage showed that while basic E linkage resulted in few false positives (PPV of 97.7%) it missed nearly 20% of matches (sensitivity of 80.7%). Further analysis also indicated that both PPV and sensitivity varied with RAC event type. In particular, links to permanent admissions were more likely to be false positives than links to respite admissions or hospital leave, and the sensitivity of the strategy was lower for admissions (under 75%) than for other RAC events (over 85%).

Given the short-comings of the basic linkage strategy in the current context – in particular, its inconsistent and relatively low sensitivity – a number of constrained E strategies were investigated to see whether using additional information for matching and/or choosing the best match could improve the quality of the E linked dataset. Again, only match passes in Table 2 with a PPV of at least 60% were retained.

Different regions (all derivable from postcode) were considered for matching to allow for reporting differences and transcription errors. Inconsistencies in reported region of usual residence were considered highly likely as a sizeable proportion of the transitions relate to people in the process of changing their usual residence (i.e. being admitted into permanent RAC for the first time) – nearly 20% of links found using basic E linkage were for permanent admissions. A number of region definitions were tested, based on two main definitions which differed primarily in the size of the region used when establishing links. The first was based on linking within SLGs (as per basic E linkage). In the second variant, matching was restricted to simple postcode-based regions, and so generally used smaller match regions.

Overall, six different versions of constrained E linkage were considered, with three based on SLG (termed SLG strategies) and three based on postcode (termed PC strategies) (see Table 4). For both the SLG and PC strategies, the three variants differed only in the last pass (pass 7 in Table 2): the first version (v1) used only either SLG region or regions defined by the full postcode or the first three digits of the postcode for matching (i.e. no pass 7); the second version (v2) matched on regions defined by the first two digits of the postcode in the final pass – essentially a metropolitan/rural breakdown for Western Australia; the third (v3) excluded two-digit postcode matching but included comparing the person's RAC facility postcode with the postcode of usual residence reported by the hospital. This last was initially considered to see if there was confusion (on the part of data providers) on what should be reported as the usual residence for people in the process of changing where they lived. That is, reporting the post-hospital rather than pre-hospital usual residence could be leading to false negatives in the matching process. Rough-guide estimates based on statistical theory in conjunction with match rates from earlier studies had indicated that, in the current context, matching within the regions used in the six strategies would lead to relatively low numbers of false matches [8].

**Table 4:** Regions used for blocking in constrained E linkage strategies

	**Pass**						
**Match strategy**	**1**	**2**	**3**	**4**	**5**	**6**	**7**

Constrained-SLG v1	SLG	SLG	SLG	SLG	SLG	3-digit PC	. .
Constrained-SLG v2	SLG	SLG	SLG	SLG	SLG	3-digit PC	2-digit PC
Constrained-SLG v3	SLG	SLG	SLG	SLG	SLG	3-digit PC	RAC PC
Constrained-PC v1	PC	3-digit PC	3-digit PC	3-digit PC	3-digit PC	3-digit PC	. .
Constrained-PC v2	PC	3-digit PC	3-digit PC	3-digit PC	3-digit PC	3-digit PC	2-digit PC
Constrained-PC v3	PC	3-digit PC	3-digit PC	3-digit PC	3-digit PC	3-digit PC	RAC PC

Note: Australian postcodes have four digits. 3-digit postcode indicates that the first three digits of the postcode were used for region matching; 2-digit postcode indicates that the first two digits of the postcode were used for region matching. In Western Australia in 2001, all postcodes had fewer than 5000 people of either sex. For 3-digit postcode regions, 85% had fewer than 2500 men aged 65+, 79% had fewer than 2500 older women with the most populous regions having fewer than 15000 older people of either sex. Among the nine two-digit postcode regions, the largest had fewer than 50000 older women and 40000 older men.

After dropping poorly performing match passes (PPV < 60%), the E linkage constrained strategies resulted in identifying between 7078 and 7595 transitions from hospital into RAC, depending on the regions used in matching (Table 5). All variants had a PPV of around 98%, but sensitivity varied between 85.6% (PC v1 strategy) and 91.5% (SLG v2, SLG v3 and PC v2 strategies). For both the SLG and PC strategies, including matching on regions defined by the first two digits of postcode (pass 7 in Table 4) increased the sensitivity of the linkage by several percentage points – the result of an additional 318 true links for the SLG version and 482 true links for the PC version (Table 5). Excluding two-digit postcode matching but allowing matching using the postcode of the RAC facility (instead of that of the person's pre-RAC usual residence) led to similar gains under the SLG strategy (320 additional true links) and significant (but somewhat fewer) gains under the PC strategy (399 additional true links compared with 482 for PC v2).

**Table 5:** PPV and sensitivity of E linkage strategies. N linkage used as the reference standard.

**Match strategy**	**True positives (a)**	**False positives (b)**	**False negatives (c)**	**Total links (E = a+b)**	**PPV (a/E)**	**Sensitivity (a/N)**
	**Number**			**Per cent**		

**Name-based N linkage**	**(N) 8106**	**. .**	**. .**	**8106**	**. .**	**. .**

**Event-based E linkage**						

Basic	6539	154	1567	6693	97.7	80.7
Constrained-SLG v1	7100	153	1006	7253	97.9	87.6
Constrained-SLG v2	7418	177	688	7595	97.7	91.5
Constrained-SLG v3	7420	170	686	7590	97.8	91.5
Constrained-PC v1	6936	142	1170	7078	98.0	85.6
Constrained-PC v2	7418	169	688	7587	97.8	91.5
Constrained-PC v3	7335	156	771	7491	97.9	90.5

As can be seen in Table 5, the basic and constrained E strategies resulted in very similar total PPVs – around 98%. However, the PPV and, in particular, the sensitivity of E links varied with both the transition type (as identified by the RAC event type) and E strategy (Table 6). Some variation was also seen in the NPV and specificity. However, these last two are not discussed in detail here as their values depend on which dataset is used to measure non-links. In summary, when compared with hospital non-links, at the total level both the NPV and specificity for all strategies were above 98%. When comparing to the RAC non-links, specificity varied less than NPV and was above 95% for all strategies and RAC event types. On the other hand, NPV varied considerably, being lowest for the basic E strategy (88%) and highest for the constrained v2 and v3 strategies (around 94%).

**Table 6:** PPV and sensitivity, by RAC event type and E linkage strategy. N linkage used as the reference standard.

**Match strategy**	**Permanent admission**	**Respite admission**	**Hospital leave**	**Social leave**	**Total**
**PPV**					
Basic	95.5	97.8	98.5	89.2	97.7
Constrained-SLG v1	95.1	98.2	98.7	95.5	97.9
Constrained-SLG v2	94.8	97.4	98.7	95.5	97.7
Constrained-SLG v3	95.1	98.1	98.6	95.5	97.8
Constrained-PC v1	95.0	98.0	98.7	99.3	98.0
Constrained-PC v2	95.0	97.2	98.7	99.3	97.8
Constrained-PC v3	95.3	97.9	98.7	99.3	97.9
**Sensitivity**					
Basic	65.2	73.8	86.4	91.9	80.7
Constrained-SLG v1	75.0	81.1	92.5	91.3	87.6
Constrained-SLG v2	88.5	86.4	93.3	91.3	91.5
Constrained-SLG v3	89.6	89.2	92.5	91.3	91.5
Constrained-PC v1	69.5	78.5	91.7	90.7	85.6
Constrained-PC v2	88.9	87.0	93.1	90.7	91.5
Constrained-PC v3	88.0	88.0	91.7	90.7	90.5
					
N links	1723	852	5370	161	8106
RAC records	4752	3297	6878	4709	19636

Note: Analysis indicated that for a small number of links the event match chosen by the N linkage strategy was not the preferred link. In particular, for 18 matches (0.2% of N links) the preferred link was to a RAC hospital leave event rather than the chosen (earlier) admission event for the same person.

In the current application, choice of region used when matching had a significant effect on the sensitivity of the linkage, but not on the PPV (Table 6). The importance in allowing for inconsistent reporting of usual residence on the two datasets when people are in transition is seen in the greater sensitivity of the strategies which allowed matching within broader regions. For example, incorporating two-digit postcode matching increased the sensitivity of the constrained SLG-based E linkage for RAC permanent admissions from 75.0% (SLG v1) to 88.5% (SLG v2). That this improvement was largely due to people moving into a RAC facility near their pre-hospital place of usual residence (rather than to transcription errors) is evidenced by the similar increase in sensitivity achieved by matching the usual residence postcode reported on the hospital data with the receiving RAC facility's postcode in pass 7 (sensitivity of 89.6% for SLG v3). The effect was smaller for respite admissions – an expected result given that most people going into respite RAC are expecting to return to their home in the community.

Choice of region had a limited effect on matches to RAC hospital leave. This is because the RAC facility is already the person's usual residence so that for RAC hospital leave the v1 and v3 variants of E linkage are the same. The difference between the sensitivity for the v1 and v2 linkages for RAC hospital leave – an increase of 42 true positives out of 43 matches for the SLG linkage – is most likely due to people going to a different, but nearby, RAC facility on discharge from hospital, with the hospital reporting the new RAC facility as the place of usual residence (i.e. reporting the 'where to' address as the usual residence rather than the 'where from' address). The postcode of the (new) receiving RAC facility was not available for this study to confirm this.

### False positives and false negatives using E linkage

Using N linkage as the reference standard, N-only links represent false negatives, while E-only links represent false positives. All link types shown in Figure 1 occurred; that is, identical links, N-only links, E-only links and mixed links. For ease of discussion, the analysis below is limited to results from the constrained SLG v2 strategy (the E linkage variant with the most links).

Overall, looking at SLG v2 linkage, there were 61 link pairs where an event in one dataset matched to a different event in the other dataset under the N and E matching strategies (before dropping the poorly performing match passes). Manual examination of these led to the following rule: if an E link matched to an event for the same person as the N link, then for the purposes of match comparison the E link was classified as a true positive. If, on the other hand, the E-link event was for a different person, then the E link was classified as a false positive.

The majority of false positives were for admissions rather than leave events (ratio of 2:1). Many of the E-only links to RAC admissions had exact matches on both date of birth and event dates, suggesting that the false matches were caused by similar hospital and/or RAC activity by similar people (in terms of recorded date of birth and sex and region of usual residence). Comparisons indicated that one of the most effective ways of reducing the number of false matches made under the E strategy would be to reduce the size of the geographic region used in matching. However, data quality and consistency issues mean that overly narrowing the geographic matching criteria would result in dropping many more true positives than false positives. For example, dropping three- and two-digit postcode matching from the SLG v1 strategy (passes 6 and 7 in Table 4) would lead to dropping 442 links, 415 of which were true matches.

Inconsistent data on the person's postcode of usual residence on the hospital and RAC datasets were the main reason for missing links to RAC admissions. This finding is confirmed by the number of additional matches made by including matching on two-digit postcode regions (v1 versus v2) and including matching which replaces the person's postcode with that of the RAC facility (v3 versus v2).

Poor region matching was a less important, but still significant, reason for missing matches among RAC leave events. However, missed matches to RAC leave events (false negatives) were primarily the result of inconsistent event dates recorded for related hospital and RAC events. The large gaps between the recorded dates for the N-linked events in these false negatives (commonly 3 or more days) indicated that it would not be possible to adjust the E matching strategy to allow capture of these matches without risking the introduction of large numbers of false positives.

Modelling the propensity of E linkage to miss N links within RAC event type, only a small number of variables were found to have statistically significant effects. Variables with significant effects in the fitted models included outlet region, hospital care type, reported post-hospital destination and whether there were leave events from hospital. Different variables were significant in the models for the three RAC event types, and in fact no statistically significant effects were found when fitting models for respite admissions. Overall, small regional differences and/or differences in basic hospital episode characteristics in the profile of N and E links to permanent RAC admission and RAC hospital leave events were detected (see Karmel and Rosman 2007 [20] for a comprehensive discussion of comparison methods and results).

### Utility of E linked datasets

Overall, when undertaking analysis of transitions, it is the combined effect of false positives and false negatives that determines the overall utility of the linked dataset. Furthermore, in addition to investigating different types of movement between the sectors, many analyses will want to compare people who have moved between the two sectors with those who have not. In this case, both linked and unlinked records are examined. Consequently, the utility of the linked data depends on whether patterns within all the various transition groups reflect the true situation.

To investigate this issue a range of typical analyses based on E and N linked datasets were compared. A number of key examples are discussed below. For E linkage, results from the PC v3 strategy were used as this strategy had high PPVs for all RAC event types (and the joint highest overall PPV), consistently high sensitivities within RAC event type and would be easier than its SLG-based counterpart to apply to national data.

### Destination after hospital

**Figure 2:**
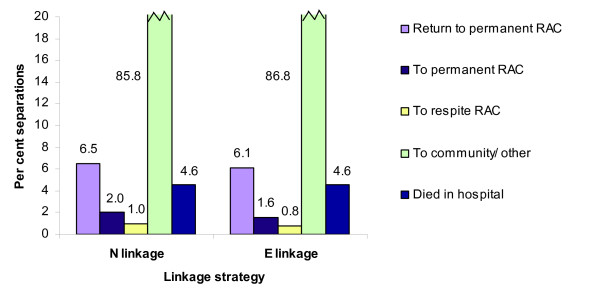
**Post-hospital destination of people aged 65 and over, by movement type, by linkage strategy**. Hospital separations are for Western Australia, 2000–01. E linkage is PC v3.

The 91% sensitivity of the E linkage strategy is reflected in the absolute numbers of people identified making the various transitions following a period in hospital (see Table 5 and Table 6). All unlinked hospital discharges, other than those ending in death, were, of necessity, classified as going 'To community/other', and as the E linkage strategy results in many more false negatives than false positives, more discharges were allocated to this residual category under E linkage than N linkage. Consequently, E linkage underestimated the number of hospital discharges relating to RAC entries. However, in proportional terms, the distribution across post-hospital destinations was very similar for the N and E linkages but with N linkage having a slightly higher proportion of discharges identified as moves from hospital to RAC (Figure 2). Even so, the relative sizes of the between-sector movement groups were similar.

### Length of stay in hospital

**Figure 3:**
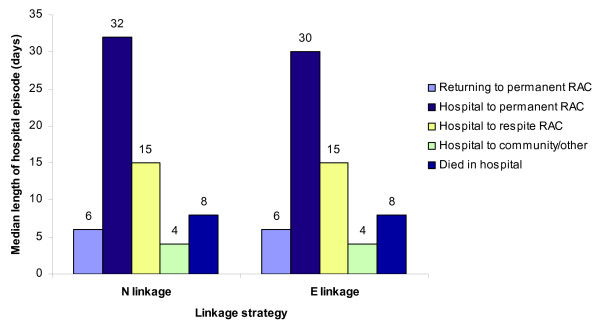
**Analysis example: Median length of hospital episode, by post-hospital destination of people aged 65+, by linkage strategy**. Hospital separations are for Western Australia, 2000–01. E linkage strategy is PC v3.

An issue that has been important in Australia for many years is whether or not older people are spending too long in hospital waiting to enter or return to a residential facility; that is, are they 'bed-blockers' [9]. Consequently, it is crucial that results from E linkage datasets reflect accurately the length of stay in hospital by post-hospital destination. Comparing median length of stay by post-hospital destination for the N and E linkage strategies showed that the strategies gave very similar results, with people who returned to the community having the shortest median hospital stay, followed by those returning to permanent RAC. Both strategies also suggested that people moving into permanent RAC have much longer median stays than other people (Figure 3).

### People with dementia

Using diagnoses reported in hospital it is possible to gain an insight into the care needs of people entering RAC with different health conditions. Consider people with and without a diagnosis of dementia in their hospital episode: both linkage strategies indicated that, irrespective of movement group, people with dementia were more likely to have high care needs than others (Table 7). In addition, the care needs differential between people with and without dementia varied considerably across the movement types, with the greatest absolute difference being for people returning to RAC after hospital leave.

**Table 7:** Analysis example: care level and dementia status for RAC entries, by transition type.

	**Name-based N linkage**					**Event-based E linkage**				
	
	**Care level**					**Care level**				
	
**Transition type**	**High**	**Low**	**All**	**Total**		**High**	**Low**	**All**	**Total**	
	**Row %**			**Col %**	**N**	**Row %**			**Col %**	**N**

**Return from hospital leave^(a)^**										

With dementia	64.5	35.5	100.0	31.0	1711	65.2	34.8	100.0	30.7	1570
Without dementia	33.6	66.4	100.0	69.0	3802	33.4	66.6	100.0	69.3	3536
*All*	*43.2*	*56.8*	*100.0*	*100.0*	*5513*	*43.1*	*56.9*	*100.0*	*100.0*	*1570*

**Into permanent RAC from hospital^(a)^**										

With dementia	83.1	16.9	100.0	45.9	769	84.3	15.7	100.0	45.2	690
Without dementia	72.6	27.4	100.0	54.1	905	74.0	26.0	100.0	54.8	837
*All*	*77.4*	*22.6*	*100.0*	*100.0*	*1674*	*78.7*	*21.3*	*100.0*	*100.0*	*1527*

**Into respite RAC from hospital^(a)^**										

With dementia	32.5	67.5	100.0	24.6	209	31.9	68.1	100.0	25.1	191
Without dementia	19.1	80.9	100.0	75.4	640	20.0	80.0	100.0	74.9	570
*All*	*22.4*	*77.6*	*100.0*	*100.0*	*849*	*23.0*	*77.0*	*100.0*	*100.0*	*761*

RAC entries are for Western Australia, 2000–01. E linkage strategy is PC v3.

(a) Based on linked hospital and RAC records.

Note: E linkage is PC v3. Diagnosis of dementia includes diagnoses of dementia and Alzheimer's disease (ICD-10-AM Edition 1 categories F00–F03, and G30) [31]. Table excludes 115 cases with missing care level, and all unlinked RAC hospital leave events.

## Discussion

The best linkages between disparate data sources are achieved using detailed personal and event information. However, there are situations in which the optimal level of detail is not available. While theoretical considerations can show whether a linkage approach is likely to result in matches with a high PPV [8], identifying strategies that give both high PPV and consistent (and, preferably, high) sensitivity across key characteristics relies largely on the availability at the development stage of a reference linkage for comparisons for testing different approaches. Often reference linkages are based on a sample of records subjected to extensive (and generally expensive) manual follow-up [26]. In the current study we were fortunate to be able to derive a comprehensive reference linkage which could be used both to measure the effectiveness of several event-based linkage strategies and to then refine them to an acceptable level of quality (false positive plus false negative error).

Our results also demonstrate the value of detailed knowledge of both service and data collection practices when building specific record linkage systems that rely on limited data. An ability to consider why there could be inconsistencies in the match data in the two datasets (for example, in address data), and subsequently to find ways to overcome these differences, is also key.

The accuracy of the constrained E linkage used in this study compares favourably with those achieved for other studies. For example when linking New Zealand census and mortality records without using name or person identifier, Blakely and colleagues reported a PPV 'in excess of 95%', with 77% of eligible mortality records being linked [11]. In addition, the preferred name-based deterministic linkage between three Canadian administrative databases had a PPV of 97–99% and a linkage rate of 88–93% (sensitivity 85–92%; [27]). However, for a particular application, whether event-based linkage is considered to be sufficiently accurate will depend on the context of the analysis. For example, in the context of testing drugs in a clinical trial it is very important that deaths be correctly identified as the future use of a drug with potential benefits – and side effects – may result. On the other hand, examination of population transitions may require less accuracy as it is the general patterns of transition that are of interest rather than outcomes for particular individuals.

Theoretical calculations of the expected number of false matches due to different people having similar demographic and event data led to PPV estimates in a similar range to those observed for the constrained linkage strategies (using formula in [8], taking into consideration the degree of match variation allowed and size of match regions). The increase in sensitivity observed as match criteria were relaxed was caused by discrepancies in both event date and demographic data in the two datasets, as evidenced by the fact that less than 54% of N links to RAC admissions matched exactly on date of birth, sex, date of transition and postcode of usual residence. The main reason for differences for postcode was inconsistent practices in reporting the address of usual residence within the hospital data. The experience of other studies has shown that improvements in data quality can greatly decrease the extent of missed links [28] and a number of developments in data collection practices since this study, particularly in recording residential region in the RAC data [29] and post-hospital destination [30], are expected to lead to reductions in both false positives and false negatives.

When looking at transition events, a linked dataset with high PPV and consistent sensitivity provides a good basis for examining transition patterns. A high PPV means that few links are false, so that the matched records provided a good basis for examining the profile of people undertaking various types of transitions between services. On the other hand, consistent sensitivity across transition categories means that the relative sizes of the groups can be gauged. Of the seven E linkage variants tested in this study, four had both high PPV and consistent sensitivity across the four movement groups from hospital into RAC identified in Table 6. All four strategies relied on auxiliary data not used explicitly in the matching. The difference between these four strategies lay solely in the region information used to establish matches.

The prevalence of false positives increased with the region population size used in the E linkage strategies (as predicted from theoretical analysis [8]). For example, links with exact matching on date of birth, sex and transition date had an overall PPV of 98.4% when matching on all four digits of the postcode exactly (PC v2 pass 1 Table 4) compared with a PPV of 94.7% for matches with correspondence only at the broad two-digit postcode region (PC v2 pass 7). In terms of population size, in the Western Australian context in 2001 regions defined by full postcode had fewer than 5000 older people (65+) for over 99% of postcodes, regions defined by the first three digits of postcode had fewer than 25000 older people (with just over 80% having fewer than 5000 people 65+) while the nine regions defined by the first two digits of postcode had populations ranging from under 5000 older people for a remote region to up to 90000 for a metropolitan region [20].

When choosing the preferred E linkage strategy to use in other studies two issues need to be considered in addition to the levels of accuracy found in the test linkage. In particular, the ease of applying the method and whether there are differences in the new population under study that need to be taken into account. In the current context, having identified several possible E linkage strategies for linking Australian hospital and RAC data we have to choose one to apply to the full national datasets for investigation into the hospital-RAC interface across Australia. In terms of ease of use, the PC methods are easier to apply than the SLG variants as derivation of SLG from postcode can be quite complex. In addition, the most accurate SLG and PC strategies had similar PPV and sensitivity; however, the SLG strategies use larger match regions and so could lead to lower PPVs in the more populous regions in other Australian states. Finally, using a particular data item (RAC facility postcode) to overcome a particular data matching issue (inconsistent reporting of usual residence) is preferred over an approach which simply relaxes match criteria (going to two-digit postcode). Taking the above into account, overall the PC v3 strategy is preferred for future analysis of the hospital-RAC interface in Australia.

All the E linkage strategies underestimated the flow of people from hospital into RAC (sensitivity 90.5% for PC v3 corresponding to a relative size of 92.4%; Table 5). While the main purpose of the comparisons with a reference linkage was to establish the accuracy of E linkage and to identify the preferred variant, the comparisons can also be used to derive estimates of the total size of the various transition groups in future analysis. That is, information on the relative size of the N and E linked datasets in our comparison can be used to adjust future E-linkage results to get overall estimates of flow from hospital into RAC in Australia. Such estimates of total flow are important for gauging the extent of the interaction between the two sectors.

## Conclusion

The current study directly compares results from linking population-wide Western Australian hospital and RAC episode data using events selected from among those linked through a person-based linkage strategy with those from a group of event-based linkage strategies. These event-based strategies (i.e. E linkages) did not use any name or unique identifier information, but relied on event details in conjunction with limited demographic data. Implementation of both these methods showed that when identifying service transition events, detailed knowledge of both the service systems and the data collection practices within those systems is essential.

Using comparisons with the name-based reference linkage, a PPV of 98% was achieved using event-based linkage and the sensitivity was greatly increased – from 81% using the simplest strategy to 91% for the preferred strategy. The event-based strategies resulted in several times more false negatives than false positives, and the volume of flow from hospital to RAC was therefore underestimated. Nevertheless, illustrative examples examining patterns of use and characteristics of people making particular types of transitions between the two sectors showed that analyses using output from the person-based N linkage and event-based E linkage strategies led to very similar conclusions. Consequently, event-based linkage provides a useful tool for examining transitions, provided there is an understanding of the limitations and caution in applying analytical techniques.

This detailed comparison of event linkage methods has also demonstrated two important roles for high quality population-based reference linkages, such as those available through the WADLS, in assisting in the development of strategies using reduced amounts of information. The first is as a tool for refinement during strategy development and the second is as a 'gold standard' against which to measure accuracy and completeness of the set of links produced and the utility of subsequent analyses.

Importantly the work reported here has allowed a 'preferred event' event-based strategy to be selected and deployed across Australia to study the national situation in movements from hospital to residential aged care facilities using databases in which detailed personal information is not available, but dates and event details can be readily accessed. More generally, the results presented here suggest that such an event-based linkage approach could be used in other areas where relatively small populations are moving across single interfaces within a narrow time window.

## Abbreviations

AIHW Australian Institute of Health and Welfare. CHIC Department of Health Western Australia Confidentiality of Health Information Committee. DEC DoHA Departmental Ethics Committee. DOB date of birth. DoHA Australian Government Department of Health and Ageing. E linkage event-based event linkage (name data not used). ICD-10-AM International Classification of Diseases 10th revision Australian Modification, based on the World Health Organization's internationally accepted classification of diseases and related health conditions. N linkage name-based event linkage. NPV negative predictive value. PC postcode. PPV positive predictive value. RAC residential aged care. SLG Statistical local group. WADLS Western Australian Data Linkage System

## Competing interests

The authors declare that they have no competing interests.

## Authors' contributions

RK undertook the event-based data linkage of transition events, refined the E strategies based on linkage comparisons, and led the analysis. DR undertook the name-based data linkage of transition events, and assisted in the analysis. Both authors read and approved the final manuscript.

## Pre-publication history

The pre-publication history for this paper can be accessed here:


